# Artificial Intelligence-Based Cervical Cancer Screening on Images Taken during Visual Inspection with Acetic Acid: A Systematic Review

**DOI:** 10.3390/diagnostics13050836

**Published:** 2023-02-22

**Authors:** Roser Viñals, Magali Jonnalagedda, Patrick Petignat, Jean-Philippe Thiran, Pierre Vassilakos

**Affiliations:** 1 Signal Processing Laboratory (LTS5), École Polytechnique Fédérale de Lausanne (EPFL), 1015 Lausanne, Switzerland; 2 EssentialTech Centre, École Polytechnique Fédérale de Lausanne (EPFL), 1015 Lausanne, Switzerland; 3 Department of Pediatrics, Gynecology and Obstetrics, Geneva University Hospitals, Boulevard de la Cluse 30, 1205 Geneva, Switzerland

**Keywords:** cervical cancer, visual inspection with acetic acid, artificial intelligence, automatic screening

## Abstract

Visual inspection with acetic acid (VIA) is one of the methods recommended by the World Health Organization for cervical cancer screening. VIA is simple and low-cost; it, however, presents high subjectivity. We conducted a systematic literature search in PubMed, Google Scholar and Scopus to identify automated algorithms for classifying images taken during VIA as negative (healthy/benign) or precancerous/cancerous. Of the 2608 studies identified, 11 met the inclusion criteria. The algorithm with the highest accuracy in each study was selected, and some of its key features were analyzed. Data analysis and comparison between the algorithms were conducted, in terms of sensitivity and specificity, ranging from 0.22 to 0.93 and 0.67 to 0.95, respectively. The quality and risk of each study were assessed following the QUADAS-2 guidelines. Artificial intelligence-based cervical cancer screening algorithms have the potential to become a key tool for supporting cervical cancer screening, especially in settings where there is a lack of healthcare infrastructure and trained personnel. The presented studies, however, assess their algorithms using small datasets of highly selected images, not reflecting whole screened populations. Large-scale testing in real conditions is required to assess the feasibility of integrating those algorithms in clinical settings.

## 1. Introduction

In 2020, cervical cancer was the fourth leading cause of death in women due to cancer and the main one in 36 countries, mostly low- and medium-income countries (LMICs) [1]. The global strategy towards the elimination of cervical cancer launched by the World Health Organization (WHO) includes early detection and screening with high-performance tests [2]. While histopathology result from biopsies is the gold standard to detect cervical lesions, it is not easily implementable worldwide. A simple and inexpensive method for screening cervical cancer is visual inspection with acetic acid (VIA). It consists of applying diluted acetic acid on the cervix and assessing the transient acetowhitening effect, which appears and disappears differently in healthy tissues, benign lesions (e.g., inflammation, metaplasia, Cervical Intraepithelial Neoplasia of grade 1—CIN1), precancerous lesions (i.e., Cervical Intraepithelial Neoplasia of grades 2 and 3—CIN2 and CIN3, respectively) and cancerous lesions. Precancerous and cancerous lesions are collectively referred to as CIN2+.

In high-income countries (HICs), VIA is performed with the assistance of a colposcope, i.e., a low-power microscope, to magnify the view of the cervix. However, those devices are rarely available for screening in LMICs due to limited financial resources, poor infrastructure and a lack of healthcare professionals. In such conditions, the assessment is usually performed with the naked eye. Visual inspection—with the naked eye or with a colposcope—is highly subjective as it entirely relies on the healthcare providers’ training and experience. Several studies suggest a high intra- and inter-observer variability with sensitivities ranging from 25.0% to 94.4% for VIA [3,4,5] and from 39% to 65% for conventional colposcopy [6,7].

Recent advances in artificial intelligence (AI) show great potential for a more objective automated detection of cervical precancer and cancer. Indeed, AI-based methods using smartphones and colposcopes images have been introduced, and yielded very promising results [8,9,10,11,12,13,14,15,16,17,18]. The current study details the results of a comprehensive systematic review aiming at identifying and comparing studies that investigate the accuracy of AI-based algorithms relying on images taken during VIA to detect precancer and cancer.

## 2. Materials and Methods

### 2.1. Protocol and Registration

The protocol has been registered in the international prospective register of systematic reviews (PROSPERO CRD42021270745) and reported in compliance with the recommendation of the Preferred Reporting Items for Systematic Reviews and Meta-Analyses (PRISMA) [19].

### 2.2. Literature Searching Strategy

A systematic literature search for publications between January 2015 and July 2022 was performed in PubMed, Google Scholar and Scopus. The search strategy included three different concepts combined using logical operators: (i) the disease (cervical cancer), (ii) the use of artificial intelligence-based algorithm (automated detection, machine learning, deep learning or artificial intelligence) and (iii) the acquisition technique (colposcope, colposcopist, colposcopic, visual inspection or acetic acid). The exact searches and results are reported in the supplementary material (Table S1).

### 2.3. Studies Selection

In order to select studies that could be further compared, the inclusion criteria were the following:

1. The studies should assess an automated artificial intelligence-based algorithm aimed at distinguishing CIN2+ (positive) from normal and benign conditions (negative), relying only on images taken during VIA. Were excluded: studies considering CIN1 as positive or not containing healthy cases.
2. For precancerous and cancerous lesions, the gold standard used to study the accuracy of the algorithms should be histopathology results, while histopathology or normal cytology and normal colposcopy were considered sufficient for negative cases. This criterion is motivated by the fact that even though histopathology results remain the most reliable method for diagnosis, some screening procedures only conduct biopsies when there is a suspicion of precancerous lesion after cytology and colposcopy. *Were excluded*: studies not mentioning their gold standards or with unclear gold standards.
3. The studies should be written in English and published between January 2015 and July 2022.
4. The studies should be original research articles and peer-reviewed. *Were excluded:* review articles, conference abstracts articles and pre-prints.

### 2.4. Data Collection and Extraction

The literature search was performed by one reviewer (RV) in PubMed and Google Scholar and by another reviewer (MC) in Scopus. The software ‘Publish or Perish’ [20] was used to collect the articles from Google Scholar, and related information was then extracted, while those could directly be extracted from PubMed and Scopus. Data from all three searches were gathered in a single spreadsheet, and duplicates were removed. Both reviewers (RV and MC) then independently assessed all publications in two stages: firstly, screening the titles/abstracts and secondly, reviewing the full texts. In case of disagreement, a third reviewer was consulted (PV).

For all included studies, the following characteristics were reported: publication details, algorithms, acquisition device, gold standard, number of patients included, dataset size and partition (training, validation, testing) and performance of the algorithms.

### 2.5. Quality Assessment

The quality of the included studies was assessed with the Quality Assessment of Diagnostic Accuracy Studies (QUADAS-2) tool [21]. Both risk of bias and applicability concerns were evaluated in terms of reference standard, index test and patient selection. In addition, flow and timing were also assessed for risk of bias only.

### 2.6. Data Analysis

The studies are highly heterogenic in terms of data acquisition method, technical approach and performance reporting. The main challenge for data analysis is raised by performance reporting: while using cross validation hinders direct comparison, most of the studies report an average accuracy, sensitivity and specificity without confidence intervals. Due to this performance reporting discrepancy, no meta-analysis could be performed.

In cases where performance metrics were not provided, but confusion matrices were, sensitivity and specificity were computed from those. When only the receiver operating characteristic (ROC) curve was provided, the following equation was used to compute the accuracy in each sensitivity and specificity measurement pair:$Accuracy\;=\;prevalence\;\cdot \;sensitivity\;+\;\left(1\;-\;prevalence\right)\;\cdot \;specificity$. Then, the sensitivity and specificity pair providing the maximum accuracy was selected for further comparison with the other algorithms.

Most of the included studies describe and compare several algorithms based on the same dataset. We thus selected the algorithm with the highest accuracy of each study for the data analysis. Sensitivity and specificity of the selected algorithms (one per included study) were reported in scattered charts. Several scattered plots were generated according to the following features: year of publication, acquisition device, dataset characteristics, algorithm family and cross validation type. Those charts directly convey visual information about the size of the dataset used (i.e., size of the dots) and the feature of interest (i.e., color code). The plots were then analyzed qualitatively.

In addition to classification techniques, the preprocessing steps of each study are briefly described and compared qualitatively.

## 3. Results

### 3.1. Study Selection

**Figure 1:**
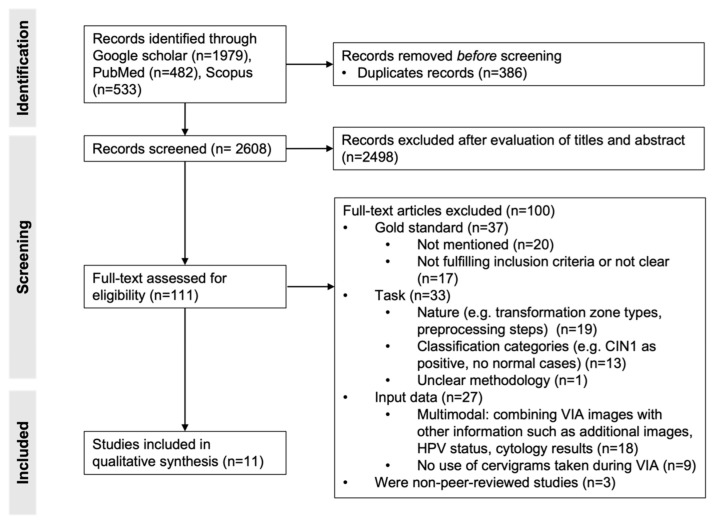
PRISMA flowchart of study selection. Adapted from [19].

We identified 482, 533 and 1979 studies in PubMed, Scopus and Google Scholar, respectively. After removal of the duplicates, 2608 publications were screened by title and abstract, which resulted in 111 studies. Full-text review of those publications led to the inclusion of 11 studies. The other 100 were mainly excluded due to mismatch between the inclusion criteria and the gold standard (n = 37), the task (n = 33), the input data (n = 27) or the absence of peer-review process (n = 3). Indeed, in some studies, the gold standard was unclear or not mentioned, not fulfilling the inclusion criteria. In others, the classification task was not the detection of cervical cancer lesions but classification of transformation zones or automatization of preprocessing steps. The classification categories could also be different (e.g., CIN1 considered as positive, no normal cases), or the methodology was unclear. While the algorithms of some studies relied on more than only VIA images (e.g., additional images, HPV status or cytology results), others did not use VIA images. The process of study selection is illustrated in Figure 1, and the 11 included studies are presented in Table 1.

### 3.2. Study Characteristics

#### 3.2.1. Data Acquisition Settings

Through a cohort in Costa Rica led by the National Cancer Institute (NCI) of the United States, some HPV and cervical cancer-related data were collected for seven years, including pictures taken during VIA. The resulting dataset (later referred to as the ‘Guanacaste dataset’) was made publicly available [22]. The images were taken using a fixed-focus, ring-lit film camera called a cerviscope. During each visit, two sequential images of the cervix after acetic acid application were taken. Several research teams built their dataset from the Guanacaste one [8,9,10,11,12].

Cho et al. [13], Peng et al. [15] and Li et al. [18] used colposcopes to acquire the images in South Korea, China and China again, respectively. All three studies collected several images per patients: while Cho et al. and Li et al. collected on average 1.8 and 3.43 images after application of acetic acid, Peng et al. acquired exactly 2 images per patient, i.e., one before applying acetic acid and one after.

Xue et al. [14], Viñals et al. [16] and Zhang et al. [17] used smartphones to collect the VIA images. Both Xue et al. [14] and Zhang et al. [17] used the MobileODT EVA system, which consists of a smartphone with magnifying lens [14], to collect images around the world (in North America, Africa, Asia and South America). In both cases, several images were acquired for each patient after the application of acetic acid. Viñals et al. [16] collected data in Cameroon and Switzerland, recording 120 images over 2 min (one frame per second), starting from the moment acetic acid was applied.

#### 3.2.2. Image Quality and Selection

Characteristics of the images, such as brightness, sharpness and colorfulness, were only described by Zhang et al. [17]. The focus of this study was the comparison of their algorithm performance when using two datasets acquired in different settings. In terms of image selection, Xue et al. [14] automatically filtered 100,000 images with a convolutional neural network (CNN)-based quality classifier. Viñals et al. [16] manually excluded images of poor quality (e.g., blurry, severe movement) or images in which lesion was not visible (e.g., excess of blood or mucus). The rest of the included studies do not detail their methodology for image selection.

#### 3.2.3. Gold Standard

Histopathology was used as the gold standard in [8,9,10,12,14,15,16,17,18]. Hu et al. [11] and Cho et al. [13] performed biopsies only when cytology and colposcopic impression after acetic acid were abnormal. The gold standard for negative cases (normal or CIN1) in those studies was thus normal cytology and colposcopy. Finally, Xue et al. [14] had histopathological reports for only a small subset of their data which we only considered for further analysis.

Even though all studies used histopathology as ground truth for positive cases, and some also for negative, most of the studies do not detail the methodology for biopsy collection, i.e., number, location and interpretation of biopsies [8,9,10,11,12,14,15,16,17,18]. Only Cho et al. [13] mention that conization biopsies were taken when there was a suspicion of precancerous lesions or cancer. In the Guanacaste dataset [8,9,10,11,12], biopsies were interpreted by multiple experts. In the study conducted by Xue et al. [14], the pathology reports consisted of a pathologist’s impression from one or more biopsies. Each report was then evaluated and categorized by a minimum of two clinicians. For the rest of the included studies [13,15,16,17,18], the methodology for biopsy interpretation was not detailed.

#### 3.2.4. Dataset Size and Partitioning

The number of patients varies considerably between studies. Three of the eleven included studies were conducted by the same research group [8,9,10] and relied on the same dataset. Those studies selected images from 1112 patients of the imbalanced Guanacaste dataset with a prevalence of 0.31 (767 negative; 345 positive). In order to create a balanced dataset of 690 cases, they randomly selected 345 cases from the negative Guanacaste ones. Xu et al. [9] report results when using both balanced and imbalanced datasets, while Xu et al. [8,10] only indicate the results on the balanced dataset. We focused our analysis on the balanced dataset only for those three studies [8,9,10] to ensure a fair comparison. All three studies used a 10-fold cross validation: in each fold, 621 and 69 patients were used for training and testing, respectively.

In [11], 9406 patients were included, from which 8917 patients were used for testing their algorithm. The selection of cases and specific images to train or validate the algorithm is not clear. They, however, conducted a comparison of automated visual evaluation performance by age groups for which the dataset sizes do not match their initial partition description. From the 8917 patients used for testing, 8689 were negative and 228 were positive, resulting in a prevalence of 0.03.

Alyafeai et al. [12] used a sub-selected balanced dataset of 348 patients from the Guanacaste one, but no information was found about the criteria of selection for the patients or images. A 10-fold cross validation was applied with a data stratified partitioning, i.e., the prevalence of 0.5 is maintained among folds. For each fold, 314 images were used for training and 34 for testing.

Cho et al. [13] selected 1426 images from 791 patients after discarding low-quality and blurred images. The prevalence of 0.72 was maintained in both training and testing sets. From each patient, the image with the highest quality was selected by two gynecologic oncologists, resulting in a final dataset containing 791 images. The use of 10-fold cross validation to develop their algorithm is unclear as it is briefly mentioned for some experiments but not for others.

Xue et al. [14] used a dataset collected by MobileODT. Gynecologic oncologists evaluated 7094 images that were used for training and testing the algorithm. In addition, the algorithm was tested on a dataset with histopathologic results, which is the only experiment of interest for the current work. For that dataset, biopsies were collected for 537 patients and 1159 images were acquired. The prevalence was 0.25 at the patient level and 0.23 at the image level. For further analysis, we will only consider their results on the data with histopathological results. All images from the same patient were assigned to the same set (either training or testing) and used independently. No use of cross validation was mentioned.

Peng et al. [15] used a balanced dataset of 300 cases resulting in a prevalence of 0.5. Each case contained a pair of images taken before and after the application of acetic acid. They used 5-fold cross validation, each fold using 240 patients for training and 60 for testing.

Viñals et al. [16] relied on a dataset of 44 patients, using 120 consecutive images after application of acetic acid per patient as input to the algorithm. From the 44 patients, 29 were positive and 15 were negative, leading to a prevalence of 0.66. A leave-one-out cross validation was done at patient level, i.e., 44 folds were done: each one using the 120 images of 43 patients for training and the 120 images of one patient for testing.

Zhang et al. [17] presented a new dataset (later referred to as ‘EVA dataset’), collected with a smartphone-based solution at different sites and thus exhibiting variability at various levels (acquisition settings, user training, use of accessories, image quality, etc.). Zhang et al. applied the same deep-learning algorithms to this dataset, as well as to the Guanacaste dataset, which were collected in well-controlled settings. In the current work, we focus on the results on the EVA dataset because when considering the Guanacaste dataset, authors might have included images from the same patient to the training and testing sets. The EVA dataset consists of 405 negative cases and 132 positive ones, with several images taken for each case, having a prevalence of 0.24. It thus results in 1027 negative images and 315 positive ones. All images from the same case were considered either in the algorithm training or testing. A 5-fold cross validation was used.

Finally, Li et al. [18] used a dataset from 732 women with several images each. From the 732 women, 375 were positive, and 357 were negative, resulting in a prevalence of 0.49. The training set consisted of 2412 images acquired from 632 women. The testing set was made of one randomly selected image for the 100 remaining women. A 4-fold cross validation was used.

Most of the included studies use single images as input to their algorithms. For instance, Cho et al. [13] acquired several images after the application of acetic acid for each participant but used only the image with the highest quality for training and testing the algorithm. Some other studies, such as Peng et al. [15] and Viñals et al. [16], relied on several images for classifying one patient. By contrast, Xue et al. [14] performed the classification at the single image level even though several images were acquired for the same patient.

#### 3.2.5. Preprocessing Steps

In the included studies, two preprocessing aspects seem to be specific to the use of VIA images: the handling of reflections caused by the speculum and mostly the detection of the region of interest (ROI), i.e., the cervix. Li et al. [18] handled specular reflections by combining smoothing and reconstruction of the images according to [23]. While Viñals et al. [16] ignored pixels above a specific threshold on their normalized amplitudes, Alyafeai et al. [12] plan to explore this aspect in future works.

Most of the detailed studies detect the region of interest as a preprocessing step of their algorithm. The methods to achieve that purpose, however, differ from manual selection to CNN-based techniques through data-driven ones. While isolation of the cervix was performed manually in Viñals et al. [16] and Li et al. [18], Cho et al. [13] assumed that the cervix is centered in the images and cropped them around that area at a given size. Peng et al. [15] segmented the cervix through k-means clustering based on location and grey information. Studies from the same authors [8,9,10] use a data-driven method: new images are compared to each labelled image of the dataset to identify which annotated image is most comparable to the unannotated one. That same ROI is then applied to the unannotated image. The rest of the studies apply a CNN-based method, either Faster R-CNN [11,14,17] or You Only Look Once [12].

Methods for preprocessing steps are highly heterogenous. Those features were thus not included in the performance analysis of the various algorithms.

#### 3.2.6. Classification Technique

Most of the studies present several algorithms, which are summarized in Table 2. The algorithm with the highest mean accuracy of each study is indicated. Through the various technical methods used, we identified four main families: (i) traditional machine learning (ML) techniques, (ii) artificial neural networks (ANN), (iii) CNN and (iv) vision transformer (ViT) combined with CNNs. The oldest studies, such as [8,9]—both published in 2015, use traditional ML techniques. The rest of the included studies rely on neural networks. Authors in [12,16] designed small neural networks for the classification task while the remaining studies adapted popular CNN architectures: AlexNet [23] was used by Xu et al. [10], Faster R-CNN [24] by Hu et al. [11] and Xue et al. [14], Inception-resnet [25] by Cho et al. [13], VGG16 [26] by Peng et al. [15] and Zhang et al. [17] and DenseNet [27] by Li et al. [18].

#### 3.2.7. Data Acquisition Settings

All the studies reported the accuracy, sensitivity and specificity of their algorithms, except [14], which provided the receiver operating characteristic (ROC) curve. As it can be observed in Table 1, the highest accuracy, sensitivity and specificity were reached by Viñals et al. [16], Hu et al. [11] and Peng et al. [15], respectively.

**Figure 2:**
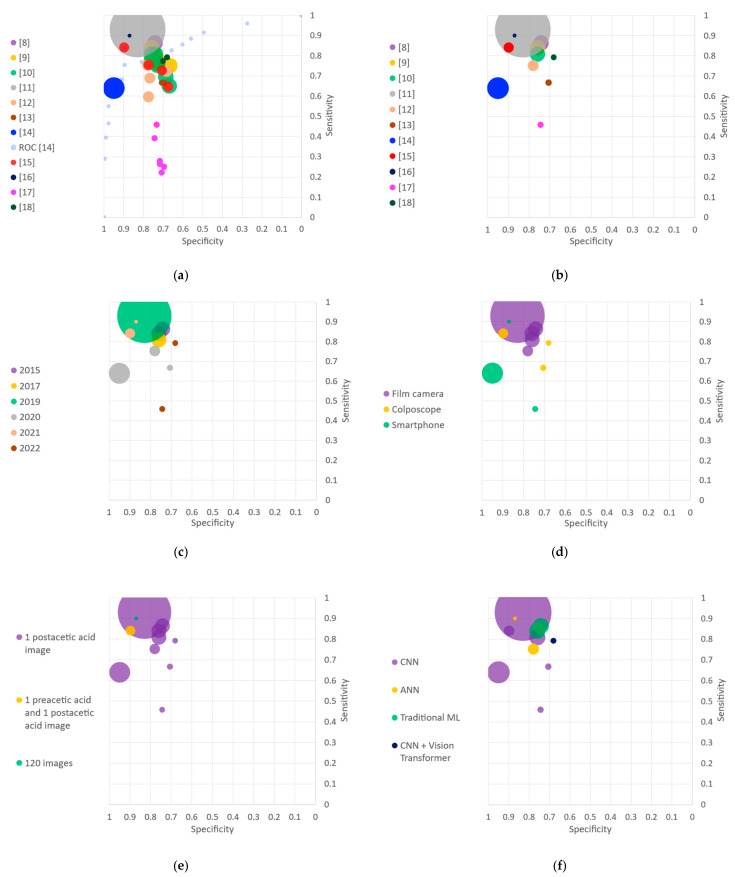

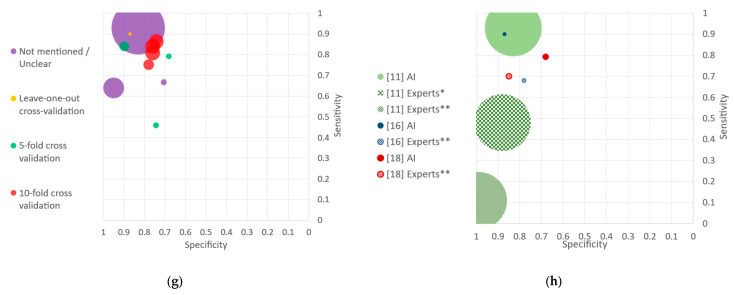
Sensitivity and specificity plots. AI: artificial Intelligence; ANN: artificial neural networks; CIN: cervical intraepithelial neoplasia; CNN: convolutional neural network; ROC: receiver operating characteristic. (**a**) Algorithms presented in the included studies with areas of the markers proportional to the sample sizes. The ROC curve of [14] is also represented. (**b**) Algorithms with the highest accuracy for each study. (**c**) Classification by year of publication. (**d**) Classification by acquisition device. (**e**) Classification by type and number of images per patient. (**f**) Classification by algorithm families. (**g**) Classification by cross validation technique. (**h**) Average experts’ performances compared to algorithms. * Classifying into normal or atypical including CIN1 and CIN2+. ** Classifying into CIN2+ and negative.

In order to allow further qualitative analysis, Figure 2 compares the various algorithms under different criteria. Each algorithm is represented by a circle whereby area is proportional to its sample size, and color indicates a characteristic of the study. Figure 2a represents the sensitivity and false positive rate—or ROC curves—for all the algorithms of each study. All algorithms from the same study are illustrated with the same color. Hu et al. [11] especially stands out by the large size of its circle—reflecting a large dataset—and high performance metrics, both in specificity and sensitivity. Regarding the studies which presented several algorithms, the diversity in performance of their different methods can be visually observed. This motivated the selection of one model per study for further analysis. Figure 2b thus presents the selected algorithm of each study, corresponding to the one with the highest accuracy.

The first criterion under which the various methods are compared is their year of publication (Figure 2c). The eleven included studies were published between 2015 and 2022: one study in 2017 and 2019, two studies in 2015, 2021 and 2022, and three studies in 2020.

The various approaches are then separated according to the device used to acquire data (Figure 2d). Three studies used a traditional colposcope, five relied on a dataset collected with a film camera, and three used a smartphone or smartphone-based device.

Regarding the input to the algorithm, almost all methods rely on single images for the classification task (Figure 2e). One study uses on a pair of images, one taken before and one after the application of acetic acid. Another study relies on 120 images, both capturing the dynamic evolution of the acetowhitening effect.

The algorithms used in the studies are separated into four families (Figure 2f). Six studies used a CNN, two studies used an ANN, two used ML traditional algorithms, and one used a ViT combined with a CNN.

Algorithms are then compared regarding their use of a cross validation technique (Figure 2g). One study used leave-one-out cross validation at the patient level, three studies used 5-fold cross validation and four studies 10-fold cross validation. For the rest of the studies, the use of cross validation was either unclear or not mentioned.

Finally, for the studies that provided a comparison of the AI algorithms with respect to the medical experts, both the algorithms and the experts’ performances are illustrated (Figure 2h). The AI algorithms systematically outperformed the medical experts.

### 3.3. Quality Assessment

**Figure 3:**
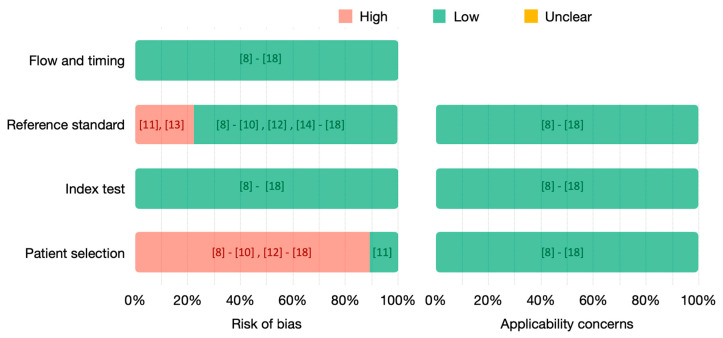
QUADAS-2 assessment of included studies. Risk of bias (**left**) and applicability concerns (**right**) are represented in percentages, and the references of the respective studies are indicated.

The quality of the included studies has been assessed using QUADAS-2 (Figure 3). We analyzed both the risk of bias and the applicability concerns due to flow and timing, reference standard, index test and patient selection.

The risk related to flow and timing and index test is low for all the included studies. Nevertheless, in ten [8,9,10,12,14,15,16,17,18] out of the eleven included studies, the risk related to patient selection is high. Indeed, small datasets are used, with highly selected images and without matching the real rate of the populations screened. The only study at low risk to introduce bias related to patient selection is [11], whose dataset includes images from more than 8000 patients with a prevalence of 0.03. Most of the included studies used histopathology as the gold standard for all the patients. However, studies [11,14] only uses histopathology as the gold standard for positive patients, having a high risk to introduce bias. In [13], only a small subset used histopathology as the gold standard. As we have focused our analysis on this subset, we consider that it has low risk. No applicability concerns were identified.

Out of the eleven studies, only [11,13] have a high risk of bias due to the gold or reference standard, using histopathology only to confirm positive patients. In [11], only patients with abnormal cytology or visual inspection were referred to colposcopy and biopsied. Thus, its dataset consists of positive cases confirmed by histology and negative cases confirmed by normal cytology and normal visual inspection. Similarly, in [13], patients with normal cytology and colposcopy were considered negative, while positive cases were confirmed by pathology. The remaining studies used histology as the gold standard for all the patients or, in [14], for a small subset used for testing. When analyzing the risk of bias introduced by the reference standard, we have not considered the biopsy collection and interpretation methodology as this was not specified in most of the studies.

## 4. Discussion

### 4.1. Dataset Size and Diversity

Among the algorithms with high specificity, three were recently published in 2020 and 2021 [14,15,16] (Figure 2c). Nevertheless, they only contain 537, 300 and 44 patients, respectively. Using such small datasets with highly selected images could give rise to a lack of generalization of the proposed methods. Further testing in larger datasets should be done to accurately estimate their performance. Similarly, we can observe that the two algorithms with the highest sensitivity also include a limited number of 44 patients [16] (see Figure 2e) or do not mention if they performed cross validation [11] (see Figure 2g), also presenting a higher risk of overfitting. By contrast, Hu et al. [11] tested its algorithm on 8917 patients and probably provided more reliable results.

Most algorithms are device-specific and are usually evaluated in very controlled settings. In some of the included studies, cross validation has been used to enhance the estimation of algorithm performance and limits overfitting. This is, however, insufficient to provide reliable generalizability for which training and testing on larger datasets are required, as well as screening populations in real conditions. Screening should indeed be conducted without high selection of the images, using different devices in different acquisition settings. The diversity of data in real conditions will raise many challenges that no one has yet considered and that are key to ensuring the success of AI algorithms.

### 4.2. Screening Approaches

Different screening approaches were used to collect the data. For instance, each screening visit from the Guanacaste dataset includes cytology, HPV testing, visual inspection with acetic acid and biopsy in some cases, resulting in a large number of patients. Hu et al. [11] used most of it, resulting in a prevalence of 0.03. The other studies relying on this dataset [8,9,10,12] have chosen a small subset of cases to develop their algorithms without specifying the criteria for the patients’ selection. Their prevalences range from 0.31 [8,9,10] to 0.5 [12].

Cho et al. [13], Xue et al. [14], Peng et al. [15], Zhang et al. [17] and Li et al. [18] did not indicate their screening approach or criteria for patient selection either. Women recruited by Viñals et al. [16] were referred for colposcopy after positive HPV testing in Cameroon and after both positive cytology and HPV testing in Switzerland. These differences in screening approach and patient recruitment highly influence the dataset in which the algorithm relies.

### 4.3. Input to the Algorithm and Preprocessing Steps

Two out of the three algorithms with the highest specificity use more than one image per patient: Peng et al. [15] relied on a pair of images taken before and after the application of acetic acid, and Viñals et al. [16] relied on 120 sequential images after the application of the acetic acid (Figure 2e). This illustrates the potential of using multiple sequential images of the cervix. Indeed, in clinical diagnosis, colposcopists not only detect the precancerous lesions based on the intensity of acetowhitening but also on time evolution. Hilal et al. [32] demonstrated that although most of the lesions are visible 1 min after the application of acetic acid, it is reasonable to perform VIA for 3 min. This suggests that instead of using single images as done in [8,9,10,11,12,13,14,17,18], relying on multiple sequential images could enhance automatic screening algorithms.

Quality and preprocessing of images can play a major role in the performance of classification algorithms. In the context of diagnosis based on VIA images, ROI detection and quality assessment of the images are essential. In addition to the different visual aspects of the cervix (e.g., transformation type, presence of lesions, blood or mucus), the VIA images may vary in terms of light, focus, position, zoom, motion, reflections or even obstruction. All those elements influence the quality of the images, and no standardized methodology considering several of these aspects was found. Also, even though the methods to isolate the cervix from the rest of the image differ among studies, the importance of detecting the region of interest is indisputable.

### 4.4. Performance Reporting

The algorithms’ performances of the included studies are presented in many ways, which complicate the comparison. Most of the studies do not mention confidence intervals nor the number of false positives, false negatives, true positives and true negatives. Those metrics can be computed from the accuracy, sensitivity and specificity values. Nevertheless, the use of cross validation without reporting the confidence intervals nor the confusion matrix prevents this computation [8,9,10,12,15,17]. The lack of confidence intervals prevents objective statistical analysis of the results and the possibility of a meta-analysis in the current work. Due to the variety in performance reported, we limited our analysis to specificity and sensitivity metrics.

Only three of the included studies—Hu et al. [11], Viñals et al. [16] and Li et al. [18]—compared their algorithms’ performances with experts assessment. For Hu et al. [11], each pair of images taken during VIA was graded by one expert, blinded from the histopathologic diagnoses, as normal, atypical, low-grade lesions, or CIN2+. For Viñals et al. [16], three experts, blinded from the histopathologic diagnoses, classified the 44 patients’ images as positive (CIN2+) or negative. Finally, for Li et al. [18], colposcopists’ diagnoses classified 100 images into four classes: negative, LSIL, HSIL and cancer. From Figure 2h, we can observe that the algorithms in the three studies achieved higher sensitivity than the experts. In Viñals et al. [16], on average, experts had worse sensitivity and specificity than the algorithm. In Hu et al. [11] and Li et al. [18], experts performed with a higher specificity than the automated algorithms.

### 4.5. Implementation for LMICs

Beyond the reliability and acceptability of computer-aided diagnostics tools, two technical key features are essential for their use in LMICs: the acquisition device and the size of the algorithm. For LMICs, portable devices seem to be more appropriate for acquiring and analyzing images than expensive tools such as colposcopes. As shown in Figure 2d, algorithms that use images from portable devices (smartphones [14,16,17] and cameras [8,9,10,11,12]) seem to achieve similar or better performance than the ones using colposcopes [13,15,18].

The use of simpler algorithms facilitates the integration into mid-range smartphones, allowing offline use of the tool. By contrast, more sophisticated algorithms might require the use of external servers for performing the classification. Figure 2f reports that traditional machine learning algorithms achieve similar performance as—or even outperform—some of the CNNs-based algorithms. For instance, among the three studies conducted by the same research group relying on the same dataset, the two studies published in 2015 [8,9] use traditional ML-based algorithms, and the one from 2017 [10] uses a CNN-based method. All three studies present similar results even when using much more sophisticated algorithms in [10] than in [8,9].

Scant evidence is available on both the usability and impact of all those studies. The assessment of the clinical effectiveness of such tools for the automated detection of cervical cancer is necessary, especially in resource-limited settings with a severe shortage of experts.

### 4.6. Limitations

Our inclusion criteria aimed at selecting comparable studies, but as a result, also restricted the scope of the analysis and introduced a selection bias. Indeed, interesting studies in terms of technical aspects might have been discarded due to the nature of their input data and ground truth.

We decided to focus on VIA only, while many research groups work on multimodal inputs. This choice could be disputed, but we consider the goal of such tools to be implemented in LMICs, which means to rely on the least information necessary in order to limit restrictions on the usage of the technology.

Also, we decided to rely on studies using only histopathology as ground truth or histopathology for positive cases and normal colposcopy and normal cytology for negative cases. However, this is comforted by the fact that histopathology remains the most widely used clinical gold standard for cervical cancer diagnosis. However, even when using histopathology as the gold standard, most studies are not precise in their methodology for biopsies collection and interpretation. This methodology is of high importance to ensure the truthfulness of the ground truth on which the automated algorithms rely. Ideally, multiple biopsies should be performed and interpreted by pathologists specialized in gynecology. CIN lesions should be reviewed independently by more than one pathologist, and the use of a two-tiered terminology (LAST) is recommended [33]. P16 immunostaining could be used as an adjunct to the diagnosis of CIN2 and to resolve diagnostic uncertainty [33].

As aforementioned, other limitations include the variety in performance reporting, the limited number of patients or images, the high selection of patients used to train and test the algorithms, or the lack of large-scale tests. As the details about the screening and recruitment methods above transpire, the risk of bias due to patient selection was high for [8,9,10,12,14,15,16,17,18] when assessing the risk with QUADAS-2. Despite the inclusion criteria regarding the gold standard, a high risk of bias remains in two of the selected studies since they do not use histopathology as the gold standard for negative patients.

## 5. Conclusions

Automated algorithms to detect precancerous and cancerous lesions using images acquired during VIA are promising, especially for countries lacking health infrastructures and where most of the deaths caused by cervical cancer occur. These images can be taken by portable devices such as smartphones or cameras, which are more accessible in LMICs than colposcopes, with no observed decrease in the performance of the algorithms.

The performance of AI algorithms using single or sequential cervical images may be as accurate as—or even better than—the human interpretation of the same images. Nevertheless, numerous limitations and constraints remain to be overcome. Most of the included studies have assessed their algorithms using small datasets, with highly selected images and without reflecting the incidence rate of the disease in screened populations. Furthermore, most of the studies do not report confidence intervals, hindering objective comparison and analysis.

In the near future, AI algorithms will probably be an essential tool in cervical cancer screening. Nevertheless, algorithms must first be generalizable, preprocessing steps optimized, and above all, tested in real-life conditions. Algorithms should be trained on larger samples with reliable histological ground truths and various populations with different acquisition procedures, including devices and settings. Proper statistical analysis of their results, demonstrating their generalizability to other populations and settings, is required to assess their potential to become the future of cervical cancer screening.

## Figures and Tables

**Table 1:** Included studies sorted by publication date.

	Study Title	First Author	Year	Acquisition Device	Cross Validation	Acquisition Country	Gold Standard	Number of Patients and Images	Number of Positive and Negative Patients
[8]	Adjustable adaboost classifier and pyramid features for image-based cervical cancer diagnosis	T Xu	2015	Film camera (Cerviscope)	10-fold cross validation	Costa Rica	**Negative:** Histopathology **Positive:** Histopathology	*Selection of balanced dataset only*	
								**Number of patients:** 690 patients **Number of images per patient:** 2 sequential images **Number of images used for classifying one patient:** 1 **Training and test sets** (sample size): 690 patients **Each fold:** 621 patients used for training and 69 for testing	345 negative (normal or CIN1) and 345 positive (CIN2+) **Prevalence:** 0.5
[9]	A new image data set and benchmark for cervical dysplasia classification evaluation	T Xu	2015	Film camera (Cerviscope)	10-fold cross validation	Costa Rica	**Negative:** Histopathology **Positive:** Histopathology	Similar to [8]	Similar to [8]
[10]	Multi-feature based benchmark for cervical dysplasia classification evaluation	T Xu	2017	Film camera (Cerviscope)	10-fold cross validation	Costa Rica	**Negative:** Histopathology **Positive:** Histopathology	Similar to [8]	Similar to [8]
[11]	An observational study of deep learning and automated evaluation of cervical images for cancer screening	L Hu	2019	Film camera (Cerviscope)	Not mentioned	Costa Rica	**Negative:** Normal cytology and normal cervicography **Positive:** Histopathology	**Number of patients:** 9406 patients **Number of images per patient:** Multiple images per patient **Number of images used for classifying one patient:** 1 **Test set** (sample size): 8917 patients **Each fold:** not applicable	Results reported on 8917 patients: 8689 negative (normal or CIN1) and 228 positive (CIN2+) **Prevalence:** 0.03
[12]	A fully-automated deep learning pipeline for cervical cancer classification	Z Alyafeai	2020	Film camera (Cerviscope)	Stratified 10-fold cross validation	Costa Rica	**Negative:** Histopathology **Positive:** Histopathology	**Number of patients:** 348 patients **Number of images per patient:** 1 image per patient **Number of images used for classifying one patient:** 1 **Training and test sets** (sample size): 348 images **Each fold:** 314 images used for training and 34 for testing	174 negative (normal or CIN1) and 174 positive (CIN2+) **Prevalence:** 0.5
[13]	Classification of cervical neoplasms on colposcopic photography using deep learning	BJ Cho	2020	Colposcope	Unclear ^a^	South Korea	**Negative:** Normal cytology and normal cervicography **Positive:** Histopathology	**Number of patients:** 791 patients **Number of images per patient:** 1.8 image per patient on average (1426 in total) **Number of images used for classifying one patient:** 1 (highest quality) **Train set**: 675 images **Test set** (sample size): 116 images **Each fold:** not applicable	**Training dataset:** 193 negative (normal or CIN1) and 482 positive (CIN2+). **Prevalence:** 0.72 **Test dataset:** 33 negative (normal or CIN1) and 83 positive (CIN2+). **Prevalence:** 0.72
[14]	A demonstration of automated visual evaluation of cervical images taken with a smartphone camera	Z Xue	2020	Smartphone	Not mentioned	Various countries in Asia, Africa, North America and South America	**Negative:** Histopathology **Positive:** Histopathology	*Subdataset of only data with histopathologic results*	
								**Number of patients:** 537 patients **Number of images per patient:** 2.2 images per patient on average (1159 in total) **Number of images used for classifying one patient:** All images are independently used **Test set (sample size):** approximately 107 cases (20% of the data are biopsy validated). **Each fold:** not applicable	**Test dataset (biopsy validated):** 405 negative cases (1027 images) (normal or CIN1) and 132 positive cases (315 images)(CIN2+). **Prevalence (at image level):** 0.23 **Prevalence (at patient level):** 0.25
[15]	Diagnosis of cervical precancerous lesions based on multimodal feature changes	G Peng	2021	Colposcope	5-fold cross validation	China	**Negative:** Histopathology **Positive:** Histopathology	**Number of patients:** 300 patients **Number of images per patient:** 2 images (preacetic and postacetic acid) **Number of images used for classifying one patient:** 1 or 2, depending on the algorithm **Training and test sets (sample size):** 300 patients **Each fold:** 240 patients used for training and 60 for testing	75 normal, 75 CIN1, 75 CIN2, and 75 CIN3 patients **Prevalence:** 0.5
[16]	Using dynamic features for automatic cervical precancer detection	R Viñals	2021	Smartphone	Leave-one-out cross validation	Cameroon and Switzerland	**Negative:** Histopathology **Positive:** Histopathology	**Number of patients:** 44 patients **Number of images per patient:** 120 sequential images **Number of images used for classifying one patient:** 120 **Training and test sets** (sample size): 44 patients **Each fold:** 43 patients used for training and 1 for testing	15 negative (12 normal and 3 CIN1) and 29 positive (11 CIN2 and 18 CIN3) **Prevalence:** 0.66
[17]	Evaluation of a new dataset for visual detection of cervical precancerous lesions	Y Zhang	2022	Eva system (smartphone with auxiliary lens)	5-fold cross validation	42 countries in North America, Africa, Asia, and Latin America		*EVA dataset* ^b^	
							**Negative:** Histopathology **Positive:** Histopathology	**Number of patients:** 544 patients **Number of images per patient:** 2.46 images per patient on average (1342 images in total) **Number of images used for classifying one patient:** 1 **Training and validation sets:** 435 patients **Each fold:** 348 for training and 87 for validation **Test set (sample size):** 109 patients	132 positive (315 images): 95 CIN2 (242 images), 32 CIN3 (67 images) and 5 cancer (6 images) 412 negative (1027 images): 206 normal (520 images) and 199 CIN1 (507 images) **Prevalence (at image level):** 0.23 **Prevalence (at patient level):** 0.24
[18]	Cervical lesion classification method based on cross-validation decision fusion method of vision transformer and DenseNet	P Li	2022	Colposcope	5-fold cross validation	China	**Negative:** Histopathology **Positive:** Histopathology	**Number of patients:** 732 patients **Number of images per patient:** 3.43 images per patient on average (2512 images in total) **Number of images used for classifying one patient:** 1 **Training and validation set:** 632 patients (2412 images) **Each fold:** 1930 images for training and 482 for validation **Test set (sample size):** 100 patients (100 images)	375 positive (1403 images): 324 CIN2+ (1223 images) and 51 cancer (180 images) 357 negative (1109 images): 175 normal (534 images) and 182 CIN1 (575 images) **Prevalence (at image level):** 0.56 **Prevalence (at patient level):** 0.49

^a^ Cross validation is mentioned but its use is not clearly explained ^b^ The other dataset (PEG) presented in that study might have included images from the same patient in both training and testing sets. For that reason, only the EVA dataset was selected and used for further analysis. CIN: Cervical intraepithelial neoplasia.

**Table 2:** Algorithms’ descriptions, images used for classification and performance.

	Classifiers	Images Used for Classification	Mean Accuracy	Mean Sensitivity	Mean Specificity
[8]	AdaBoost classifier. Multi-feature descriptors are used combining a pyramid histogram of oriented gradients (PHOG), the pyramid color histogram in L∗A∗ B space (PLAB) and the pyramid histogram of local binary pattern (PLBP) ^a^	1 postacetic acid image	0.803	0.864	0.742
[9]	7 models are proposed ^a^				
✔	(i) Random forest (RF)	1 postacetic acid image	0.800	0.841	0.759
	(ii) Gradient boosting decision tree (GBDT)	1 postacetic acid image	0.786	0.820	0.751
	(iii) AdaBoost	1 postacetic acid image	0.768	0.777	0.759
	(iv) Support vector machines (SVM)	1 postacetic acid image	0.748	0.765	0.730
	(v) Logistic regression (LR)	1 postacetic acid image	0.742	0.762	0.722
	(vi) Multilayer perceptron (MLP)	1 postacetic acid image	0.753	0.778	0.728
	(vii) k-Nearest Neighbors (kNN)	1 postacetic acid image	0.709	0.751	0.667
[10]	Several classifiers are analyzed. The sensitivity, specificity and accuracy are only specified for few of them ^a^				
✔	(i) Fine-tuned CaffeNet-based CNN (network adapted from Alexnet [24] with softmax classifier pretrained with ImageNet	1 postacetic acid image	0.784	0.809	0.759
	(ii) Support vector machines (SVM) using hand-crafter pyramidal features (PLBP, PLAB and PHOG).	1 postacetic acid image	0.772	0.786	0.758
	(iii) Support vector machines (SVM) using features extracted with a CaffeNet (network adapted from Alexnet [24]) and one fully-connected layer	1 postacetic acid image	0.660	0.651	0.670
	(iv) Support vector machines (SVM) using features extracted with a CaffeNet (network adapted from Alexnet [24]) and two fully-connected layers	1 postacetic acid image	0.691	0.696	0.687
	(v) Fined-tuned support vector machines (SVM) using features extracted with a CaffeNet and one fully-connected layer	1 postacetic acid image	0.742	0.754	0.730
	(vi) Fined-tuned support vector machines (SVM) using features extracted with a CaffeNet and two fully-connected layers	1 postacetic acid image	0.746	0.765	0.728
	(vii) Fined-tuned AdaBoost classifier using features extracted with a CaffeNet and two fully-connected layers	1 postacetic acid image	0.774	0.809	0.739
[11]	Faster R-CNN architecture [25].	1 postacetic acid image	0.832	**0.930**	0.830
[12]	4 different models.				
	(i) CNN with two convolutional layers. Automatic feature extraction.	1 postacetic acid image	0.682	0.597	0.774
	(ii) CNN with three convolutional layers. Automatic feature extraction.	1 postacetic acid image	0.703	0.723	0.683
	(iii) ANN with one hidden layer. Hand-crafted features.	1 postacetic acid image	0.729	0.690	0.768
✔	(iv) ANN with two hidden layers. Hand-crafted features.	1 postacetic acid image	0.771	0.752	0.780
[13]	2 networks used.				
✔	(i) Inception-Resnet-v2 (modified version of the Inception-v3 model) [26]	1 postacetic acid image	0.693	0.667	0.706
	(ii) Resnet-152 (updated version of the Resnet model) [27]	1 postacetic acid image	0.689	0.667	0.699
[14]	Faster R-CNN architecture [25] ^b^.	1 postacetic acid image	AUC = 0.87 (95% CI 0.81–0.92)		
[15]	Algorithms based on VGG16 [28]				
	(i) Network model that uses VGG16 to extract the features of postacetic acid test colposcopy images	1 postacetic acid image	0.660	0.647	0.675
	(ii) Network model that uses VGG16 to extract the features of preacetic acid test and postacetic acid test colposcopy images	1 preacetic acid and 1 postacetic acid image	0.717	0.728	0.707
	(iii) Network model that uses VGG16 to extract the features of registered preacetic acid test and postacetic acid test colposcopy images	1 preacetic acid and 1 postacetic acid image	0.767	0.755	0.774
✔	(iv) Network model that uses VGG16 to extract the features of registered preacetic acid test and postacetic acid test cervical images (after cervical region extraction).	1 preacetic acid and 1 postacetic acid image	0.863	0.841	**0.898**
[16]	Pixel-wise classification using an ANN with one hidden layer and combined with region growing segmentation.	120 images during VIA	**0.886**	0.897	0.867
[17]	3 models are proposed.				
	(i) LeNet [29]	1 postacetic acid image	0.609	0.265	0.717
	(ii) CaffeNet (network adapted from Alexnet [24])	1 postacetic acid image	0.624	0.280	0.733
	(iii) Pretrained VGG16 [28] trained on				
✔	(a) 16 top layers	1 postacetic acid image	0.676	0.459	0.744
	(b) 12 top layers	1 postacetic acid image	0.632	0.392	0.708
	(c) 8 top layers	1 postacetic acid image	0.582	0.223	0.696
	(d) 4 top layers	1 postacetic acid image	0.585	0.251	0.691
[18]	2 models are proposed.				
	(i) DenseNet161 [30] with EfficientNetV2 [31]	1 postacetic acid image	0.740	0.774	0.702
✔	(ii) DenseNet161 [30] with ViT	1 postacetic acid image	0.740	0.792	0.681

✔ Selected as representative algorithm of the study (higher accuracy). ^a^ Balanced dataset. ^b^ Subset of the data with histopathologic diagnoses. ANN: artificial neural network; CI: confidence interval; CNN: convolutional neural network; GBDT: gradient boosting decision tree; kNN: k-Nearest Neighbors; LR: logistic regression; MLP: multilayer perceptron; PHOG: pyramid histogram of oriented gradients; PLAB: pyramid color histogram in L∗A∗B space; PLBP: pyramid histogram of local binary pattern; RF: Random forest; characteristic; ROC: receiver operating characteristic; SVM: support vector machine; VIA: visual inspection with acetic acid; ViT: Vision transformer.

## Data Availability

The datasets used and/or analyzed during the current study are available from the corresponding author on reasonable request.

## Supplementary Materials

The following supporting information can be downloaded at: https://www.mdpi.com/article/10.3390/diagnostics13050836/s1, Table S1: Searches and results.
