# Supplementary material for: Artificial Intelligence-Based Cervical Cancer Screening on Images Taken during Visual Inspection with Acetic Acid: A Systematic Review
Source: Diagnostics (Basel). 2023 Feb 22;13(5):836. doi: 10.3390/diagnostics13050836 (PMC10001377; doi:10.3390/diagnostics13050836)
Supplement: Supplementary file 1 [file diagnostics-13-00836-s001.zip › diagnostics-2181648-supplementary.pdf]

Additional File 1.

Table S1. Searches and results

Search engine	Initial search strategy	Number of articles
Google Scholar	cervical cancer AND (automatic detection OR machine learning OR deep learning OR artificial intelligence) AND (colposcope OR colposcopist OR colposcopic OR visual assesment OR visual inspection OR VIA)	1979
PubMed	cervical cancer AND (machine learning OR deep learning OR artificial intelligence OR automatic detection)	482
Scopus	cervical cancer AND (machine learning OR deep learning OR artificial intelligence OR neural network)	533
